# Epoxy/Layered Double Hydroxide Nanocomposites: Investigating the Influence of Preparation Method on Mechanical Properties

**DOI:** 10.3390/polym18121444

**Published:** 2026-06-09

**Authors:** Daiva Zeleniakiene, Kristina Zukiene, Stanislav Stankevich, Petr Knotek, Claudia M. Rocha, Jakub Oprsal, Rochele Pinto, Sigitas Kilikevičius, Cristina Neves, João Tedim

**Affiliations:** 1 Department of Mechanical Engineering, Kaunas University of Technology, Studentu St. 56, 51424 Kaunas, Lithuania; 2 Department of Production Engineering, Kaunas University of Technology, Studentu St. 56, 51424 Kaunas, Lithuania; 3 Institute for Mechanics of Materials, University of Latvia, Jelgavas St. 3, LV-1004 Riga, Latvia; 4 Department of General and Inorganic Chemistry, University of Pardubice, Studentska 573, 532 10 Pardubice, Czech Republic; 5 Smallmatek—Small Materials and Technologies, Lda, Rua dos Canhas, 3810-075 Aveiro, Portugal; 6 SYNPO a.s., S. K. Neumanna 1316, 532 07 Pardubice, Czech Republic; 7 Department of Transport Engineering, Kaunas University of Technology, Studentu St. 56, 51424 Kaunas, Lithuania; 8 DEMaC-Aveiro Institute of Materials, University of Aveiro, Campus Universitário de Santiago, 3810-193 Aveiro, Portugal

**Keywords:** layered double hydroxides, epoxy, nanocomposite, mechanical properties

## Abstract

This study investigates the macro- and micromechanical properties of epoxy-based nanocomposites containing Mg-Al/NO_3_ layered double hydroxides (LDHs), with a comparative evaluation of two preparation routes: solvent exchange and direct drying from an aqueous LDH slurry. LDHs were characterised using atomic force microscopy (AFM), scanning transmission electron microscopy, and X-ray diffraction methods. Tensile tests and AFM nanomechanical mapping were used to obtain the macro- and micromechanical properties of LDH/epoxy nanocomposites, respectively. Both preparation methods resulted in nanocomposites with comparable mechanical performance. Young’s modulus increased by approximately 30–70% at lower LDH loadings (1–2 wt.%), and the ultimate tensile strength remained largely unchanged compared to pure epoxy. At higher LDH contents, tensile strength decreased by approximately 15–26%, while fracture strain decreased by up to 54%, which was attributed to particle aggregation, as confirmed by scanning electron microscopy and energy-dispersive X-ray spectroscopy. The mechanical response and stiffness maps obtained by the peak force AFM nanomechanical mapping revealed that nanocomposites prepared using solvent-exchanged LDHs exhibit significantly narrower interfacial widths and higher stiffness compared to those made by the conventional approach, indicating improved interfacial bonding and mechanical performance. Overall, the solvent-exchange approach proved effective for improving the interfacial properties and stiffness of epoxy/LDH nanocomposites.

## 1. Introduction

Layered double hydroxides (LDHs), also known as hydrotalcite-like clays, are two-dimensional layered nanomaterials consisting of positively charged, mixed metal hydroxides, intercalated with exchangeable anions and water molecules [1]. LDHs are considered environmentally benign materials due to their relatively low toxicity, good biocompatibility, and ability to function as halogen-free flame retardants and corrosion-protective systems [2]. In addition, LDHs can contribute to the development of safer and more sustainable polymer composites by enabling the incorporation of multifunctional protective and environmentally oriented properties into materials [3]. LDHs serve as promising materials in applications such as adsorbents [4], catalysts [5,6,7], nanocontainers for corrosion inhibitors [8,9], flame retardant additives [10], nanocomposites [11], and as drug delivery systems [12], among others.

Experimental investigations on polymer/LDH nanocomposites have shown that the exfoliation of bulky, solid LDHs is a critical point for improving the mechanical [13], thermal [14,15], electrical [16], and magnetic properties [17] of the final composite. However, the exfoliation of LDHs in solvent is difficult, owing to their high charge density on the layers. To obtain high performance LDH-based nanocomposites, their homogeneous dispersion into the polymer matrix is imperative. The incorporation of LDHs into polymer matrices can be carried out in various ways: in situ polymerisation, direct intercalation of the polymer in-between LDH layers, and template synthesis [15].

More recently, alternative preparation approaches such as solvent-assisted delamination [18], surface modification [19], and aqueous miscible organic solvent treatment [20] have been increasingly investigated to improve LDH dispersion, suppress particle restacking, and enhance interfacial compatibility with polymer matrices. The use of LDHs as nanofillers in polymers, particularly in epoxy-based composites, has been progressively investigated [21]. However, the incorporation of hydrophilic LDHs into highly hydrophobic polymer matrices requires modification of the LDHs. The use of anionic surfactants such as sulfonates [22] increases the interlayer spacing and improves hydrophobic surface properties [23]. Silane modification is another method through which the compatibility and hydrophobicity of LDHs can be improved to be incorporated in non-polar mixtures [24].

Inclusion of exfoliated LDHs into polymer matrices, such as epoxy resins, at smaller concentrations, has shown beneficial effects on nanocomposite properties. In particular, the presence of Co-Al LDHs in polyamide 6 (PA6) matrix enhanced the tensile strength and modulus with increasing concentration of LDHs and was significantly higher than that of neat PA6 [25]. The presence of LDHs also improved the hardness of the composite by 35% at 2 wt.% of Co-Al LDHs when compared to the neat PA6 samples. The use of organo-modified Mg-Al LDHs in an epoxy-based fibre metal laminate reinforced with E-glass improved the hardness, flexural, tensile, and impact strength of the composite [26]. In an investigation carried out on polyethylene/Mg-Al LDHs-based polymer nanocomposites, it was observed that various forms of LDHs were present in the matrix, from exfoliated nano-sized particles to aggregated clusters. The clusters exhibited crack-arresting behaviour due to efficient transfer of stress from the matrix to the particle [27].

In the case of polypropylene/LDHs nanocomposites, thermal stability, tensile modulus, storage modulus and ability to retard flammability were improved by the addition of organo-modified Mg-Al LDHs [28,29,30]. The effect of LDHs on Bisphenol A epoxy resins has been investigated, where Mg-Al LDHs were intercalated with dodecylsulfate (DS) and then incorporated into the epoxy resin. The highest tensile strength was observed for 3 wt.% of LDHs and highest flexural strength at 1 wt.% [31]. Remarkably, using a silane coupling agent, DS-modified Mg-Al LDHs/Bisphenol A epoxy nanocomposites exhibited an even higher improvement of 50.8% in Young’s modulus and 90% in flexural modulus at no more than 0.25 wt.% loading of LDHs. This could be attributed to the enhanced interfacial interaction by silane functionalisation, additionally reflected in the improvement in thermal stability [32].

Although numerous studies have demonstrated that LDHs can improve the mechanical and multifunctional properties of polymer nanocomposites, most investigations have primarily focused on filler composition, surface functionalisation, and LDH loading. Comparatively less attention has been paid to the influence of LDH post-processing routes on dispersion quality and interfacial interactions within epoxy matrices. In particular, the effect of solvent-exchange treatment compared with conventional drying of aqueous LDH suspensions on particle morphology, dispersion state, and the resulting mechanical behaviour of epoxy/LDHs nanocomposites remains insufficiently understood.

The primary goal of the present work was to fabricate epoxy/Mg-Al/NO3 LDHs nanocomposites and investigate the mechanical properties of the resulting systems, using two types of LDHs materials. Starting from LDHs obtained by co-precipitation in aqueous slurry form, LDHs were post-processed by consecutively replacing the solvent using the so-called solvent-exchange method or, alternatively, dried in the oven. The solvent-exchange route was selected because solvent-assisted processing of layered materials has been reported to facilitate partial delamination and exfoliation by reducing interlayer interactions and suppressing particle restacking during drying [33,34]. Therefore, the extent of LDH exfoliation was also investigated.

Typically, LDH samples obtained by co-precipitation and ion-exchange [1,35] are aqueous slurries with good dispersion ability in water-based polymeric formulations. However, when powders need to be prepared because of problems associated with the presence of water, the direct removal of water by drying leads to aggregation of individual LDH particles. The approach proposed in the present work aims to evaluate whether solvent-exchange processing can reduce the issues associated with drying-induced aggregation, focusing instead on the use of solvent-based LDH slurry. By evaluating this processing method, the research seeks to determine how the mechanical properties of the resulting nanocomposites are influenced. Such an investigation is critical in the quest for sustainable materials that meet or exceed the performance of traditional options, thereby contributing to the advancement of eco-friendly composite technologies.

## 2. Materials and Methods

### 2.1. Materials

Mg-Al/NO_3_ LDHs with a molar Mg:Al ratio of 2:1 were used in two forms: a slurry in water (containing 15–20 wt.% solids, with an average particle size of 400–500 nm) and powder (with particle size smaller than 20 µm), both provided by Smallmatek Lda (https://www.smallmatek.pt/, accessed on 5 June 2026), Aveiro, Portugal.

A two-component epoxy system was used as the polymer matrix, consisting of bisphenol A epoxy resin CHS 582 and isophorone diamine hardener Telalit 0420, both supplied by Synpo a.s. (https://www.synpo.cz/en, accessed on 5 June 2026), Pardubice, Czech Republic, in a 4:1 weight ratio.

As solvents for the particle washing and exfoliation procedure of the LDH slurry in water, 96% ethanol (Aga, Ph. Eur), ≥99% acetone (VWR, Technical Grade, VWR International BV, Leuven, Belgium), and 99% xylene (Fisher Scientific, ACS reagent, Loughborough, UK) were used.

Disperbyk 180, supplied by BYK Additives & Instruments (Wesel, Germany) was used as a dispersant for the LDH powder particles. Disperbyk 180 is an alkylolammonium salt of a copolymer with acidic groups.

### 2.2. Preparation of Epoxy/LDH Nanocomposite Samples

**Figure 1:**
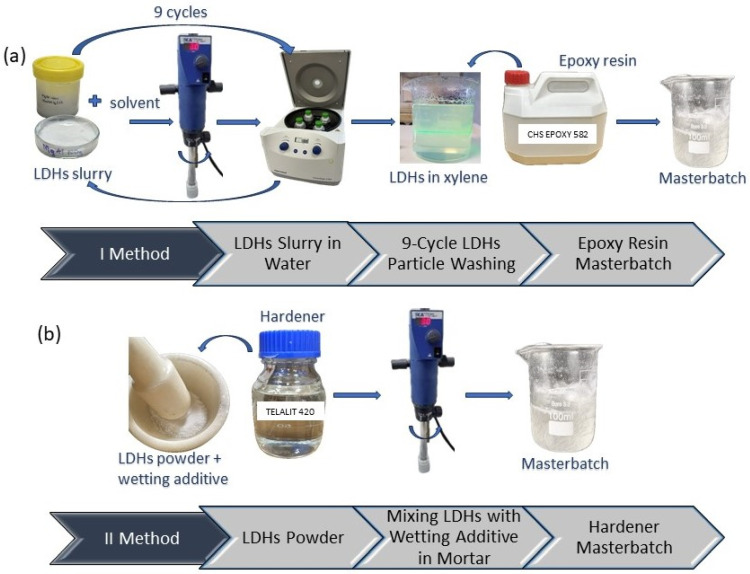
LDH masterbatch preparation methods based on the utilisation of: LDH slurry in water and gradual substitution for organic solvents (**a**); LDH powder with wetting additive (**b**).

Two different methods for preparing LDH masterbatches were developed and used in the production of epoxy/LDH nanocomposite samples (Figure 1).

In the first method, the LDH slurry in water was selected as the starting material because there was a desire to use a product with smaller particle size and prevent aggregation of LDHs upon drying (Figure 1a). A nine-cycle washing procedure was utilised for the LDH particles, progressively replacing water with ethanol, followed by acetone, and xylene at the end. This procedure also aimed to delaminate and exfoliate relatively large LDH particles in slurry into the nanoparticle size range. The LDH slurry in water was mixed with ethanol at a ratio of 1:4 (slurry to ethanol) by a high-shear mixer ULTRA TURRAX^®^ T18 (IKA, Staufen, Germany) at 2100 rpm for 15 min, followed by centrifugation (Z446, Hermle Labortechnik GmbH, Wehingen, Germany) at 6000 rpm (5830 g) for 15 min. After centrifugation, the supernatant was discarded, and additional ethanol was poured, maintaining the same slurry-to-ethanol ratio. This cycle of ethanol addition and centrifugation was conducted two more times, making a total of three repetitions. The procedure was then repeated under identical conditions, except for the washing solvents. Acetone was utilised for the fourth through sixth cycles, while xylene was used for the seventh through ninth cycles.

A notable change in the suspension’s appearance from a matte to gel-like consistency occurred during the seventh washing when acetone was replaced with xylene. The Tyndall effect, observed in the resulting material, indicated the presence of fine particles in this gel-like suspension. After the final centrifugation, the LDH particles suspended in the gel-like xylene solution were retrieved from the centrifuge tubes. The content of LDH particles in this solution was measured using thermal gravimetric analysis (TGA Q500, TA Instruments, New Castle, DE, USA) and a gravimetric method by allowing natural evaporation of xylene in a well-ventilated fume hood at room temperature for 24 h. Both methods yielded identical results, revealing that the LDH content in the gel-like solution ranged from 23 to 26 wt.%, depending on the batch. The final washing cycle was conducted and the resulting mixture was utilised as the masterbatch. The LDH in xylene gel was mixed using a high-shear mixer for 15 min at 15,000 rpm with the required amount of epoxy resin CHS 582 in an ice bath to prevent overheating. The mixing was completed with an ultrasonic probe at 40% amplitude, using a 12 mm Ti-tip (VCX 500, Sonics & Materials, Newtown, CT, USA) for 5 min, with a one-minute pause after each minute in the ice bath. The LDH content in the masterbatch was 8 wt.%.

The second method for the preparation of the LDH masterbatch utilised LDH particles in powder form (Figure 1b). The LDH powder was placed in a mortar and ground for 15 min to obtain a finer texture. To ensure a higher-quality dispersion of LDH particles within the hardener, a dispersant, Disperbyk 180, was then added to the mortar at a ratio of 2.3:1 and mixed for an additional 10 min. Subsequently, this paste was blended with hardener Telalit 0420 in the mortar to prepare a well-dispersed thick mixture. The paste was further mixed with a high-shear mixer for 15 min at 15,000 rpm with the required amount of hardener in the ice bath to prevent overheating. The mixture was then subjected to vacuum for 30 min. The LDH content obtained in the masterbatch was 21 wt.%.

Epoxy resin masterbatches were diluted with a specific amount of epoxy resin to achieve LDH particle contents of 1, 2, and 5 wt.% in the final nanocomposite. Similarly, the hardener masterbatch was diluted with a specific amount of hardener. The epoxy resin was mixed with the hardener at a weight ratio of 4:1, the mixture was manually stirred for 10 min and then degassed using vacuum.

It should be noted that the two preparation routes were designed to represent practically relevant processing approaches rather than a single-variable model experiment. In Method I, the solvent-exchanged LDH/xylene gel was incorporated into the epoxy resin because this route provided better compatibility with the organic resin phase. In Method II, dried LDH powder was incorporated into the hardener together with Disperbyk 180, since preliminary processing without a wetting/dispersing additive did not provide a sufficiently homogeneous dispersion. The use of Disperbyk 180 therefore reflects the conventional practical route required for processing dried LDH powder. In all cases, the final epoxy-to-hardener ratio was kept constant at 4:1 by weight, and the same curing and post-curing conditions were applied.

A total of six groups of epoxy/LDH composite specimens were prepared, along with an additional group of pure epoxy samples serving as a reference. Three groups of epoxy/LDH specimens for each method were prepared with LDH contents of 1, 2, and 5 wt.%, respectively. The sample coding is presented in Table 1. Each group consisted of six specimens. Dog-bone shaped specimens, with a gauge section cross-sectional area of 3 × 1 mm according to ISO 527 standard [36], were moulded using silicone moulds. All samples were maintained at room temperature for 24 h, followed by post-curing in an oven for 2 h at 60 °C, 1 h at 80 °C, and 1 h at 120 °C.

### 2.3. Materials Characterisation and Testing Methods

Since the nine-cycle particle washing procedure could exfoliate relatively large LDH particles present in a slurry, it was necessary to ascertain the size and confirm whether the LDH particles in the resulting gel-type solution in xylene were still layered. The LDH particle height and lateral size were measured using atomic force microscopy (AFM) (Dimension Icon, Bruker, Billerica, MA, USA). As the LDHs in xylene were in liquid form, samples for AFM observations were prepared by spin-coating onto a mica substrate and drying at room temperature [37]. The surface topography of the cryo-cut composite was examined in PeakForce quantitative nanoscale mechanical mode with a spring constant of 0.4 N·m^−1^, employing ScanAsyst-Air tip (Bruker, Billerica, MA, USA), for details of cryo-cut preparation, measuring and analysis [38].

The morphology of the materials was also assessed by transmission electron microscopy using a STEM Hitachi HD2700 (Hitachi High-Technologies, Tokyo, Japan), operated at 200 kV. The samples were prepared by dropping a dispersion of the slurry in ethanol onto a carbon-coated copper grid and leaving it to dry in air.

The X-ray diffraction (XRD) patterns of two LDH samples (LDHs in xylene (dried) and in powder form) were obtained using a D8 Advance diffractometer (Bruker, Karlsruhe, Germany) set up in the Bragg–Brentano configuration. The instrument utilised CuKα radiation (λ = 1.5418 Å), with a tube voltage of 40 kV and a current of 40 mA, filtered by a Ni 0.02 filter. Data collection spanned a 2θ range of 3° to 70°, with a step size of 0.02° and a counting time of 96 s per step. The measurements were conducted with a Bruker LynxEye fast counting detector and a coupled two theta/theta scan mode.

Fourier-transform infrared (FTIR) spectroscopy was used to investigate the chemical composition and structural characteristics of epoxy/LDH nanocomposites prepared via two different methods. FTIR spectra were collected using a PerkinElmer spectrometer Spectrum Two (PerkinElmer, Waltham, MA, USA) with a UATR TWO unit (diamond), 64 scans, 4 cm^−1^ resolution, in a wavelength range of 400–4000 cm^−1^.

The tensile tests of epoxy/LDH nanocomposites were performed using a H10 KT universal column testing machine (Tinius Olsen, Salfords, UK), with a crosshead strain rate of 2 mm/min.

The fracture surfaces of epoxy/LDH samples were analysed using scanning electron microscopy (SEM) (S-3400N, Hitachi, Japan). SEM images were captured under high vacuum at an accelerating voltage of 5 keV, with magnifications ranging from 500× to 5000×, using a BRUKER Quantax EDS detector (Bruker Nano GmbH, Berlin, Germany).

Using the same S-3400N scanning electron microscope in conjunction with a Bruker Quad 5040 (Bruker, Karlsruhe, Germany) energy-dispersive X-ray spectroscopy (EDS) system, the samples were examined for elemental analysis. Measurements were performed utilising a secondary electron detector, with the SEM operating at an accelerating voltage of 15 keV.

**Figure 2:**
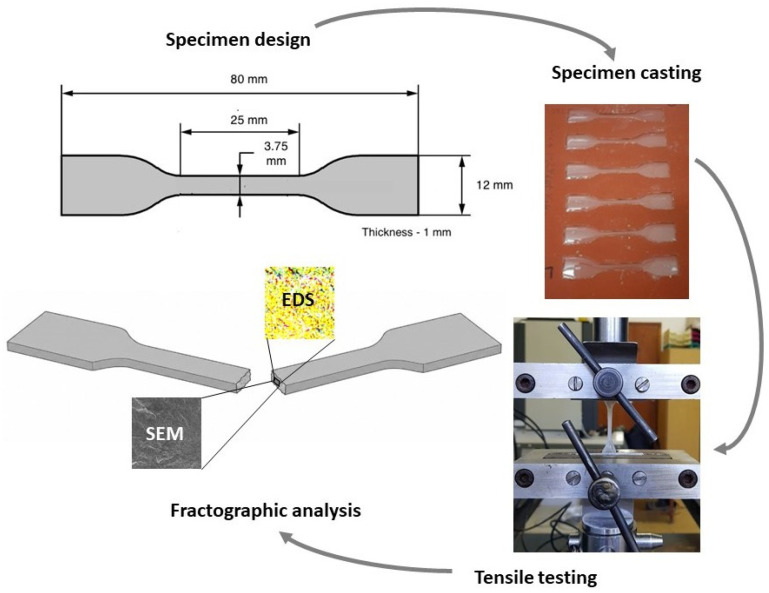
Schematic illustration of the experimental workflow used for the evaluation of macromechanical properties and fracture surface analysis of epoxy/LDH nanocomposites.

A schematic overview of the tensile specimen preparation, testing, and fracture surface analysis workflow used in the present study is presented in Figure 2.

## 3. Results

### 3.1. Characterisation of LDHs Nanoparticles

**Figure 3:**
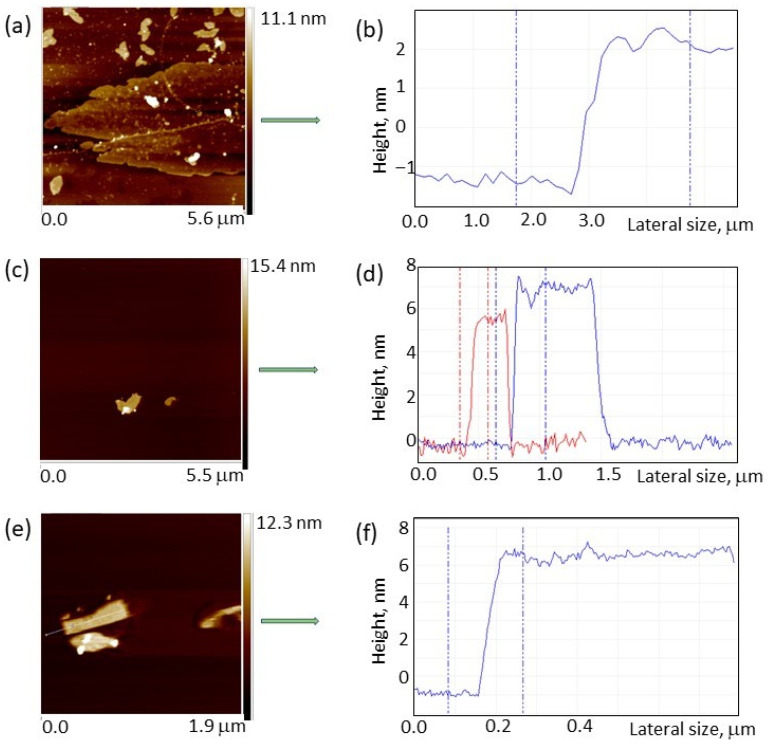
AFM analysis: AFM topographical images (**a**,**c**,**e**) and corresponding height profiles (**b**,**d**,**f**) of different LDH particles obtained by the solvent-exchange method.

AFM analysis of dried solvent-exchanged LDHs showed that particles had a lamellar structure without the detectable presence of xylene (Figure 3). Samples included different types of particles: lamellae with a height of about 3 nm and lateral size (width) over 5 µm (Figure 3a,b); fragments with a height 3–10 nm and a width < 1 µm were also observed (Figure 3c–f). LDH particles were still layered. Taking into account the reported thickness range of a single LDH layer of 0.68–1.13 nm, as reported by Zhang Y. et al. [33], and assuming a single layer thickness of approximately 0.7 nm, the LDH nanoflake in xylene was composed of 4 to 13 layers. The aspect ratio was remarkably high, exceeding 1000.

**Figure 4:**
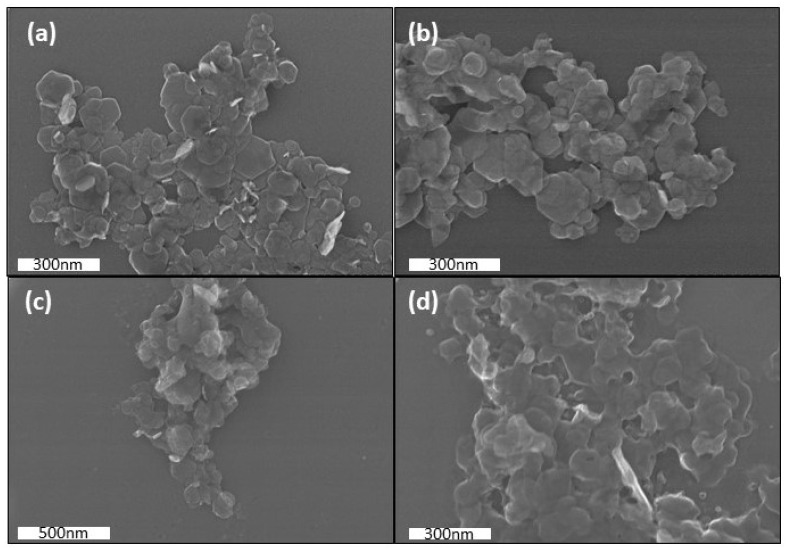
STEM images of LDH particles obtained by the solvent-exchange method: (**a**) before and (**b**) after the solvent-exchange process for the fraction recovered from the bottom of the centrifugation tubes, and (**c**,**d**) for the fraction remaining in the supernatant.

STEM images acquired before and after the solvent-exchange process reveal LDH nanoplates are similar in size and shape (Figure 4). It is worth noting that the LDH sample analysed was the fraction recovered from the bottom of the tubes after centrifugation, which was subsequently used in coating preparation. The LDH fraction that remained in the supernatant revealed less defined lamellae, irregular and fragmented structures, which could indicate some level of exfoliation. Therefore, although some level of exfoliation and particle fragmentation may have occurred, most of the LDHs that were used in coating preparation remained similar to the initial aqueous-based LDH particles.

**Figure 5:**
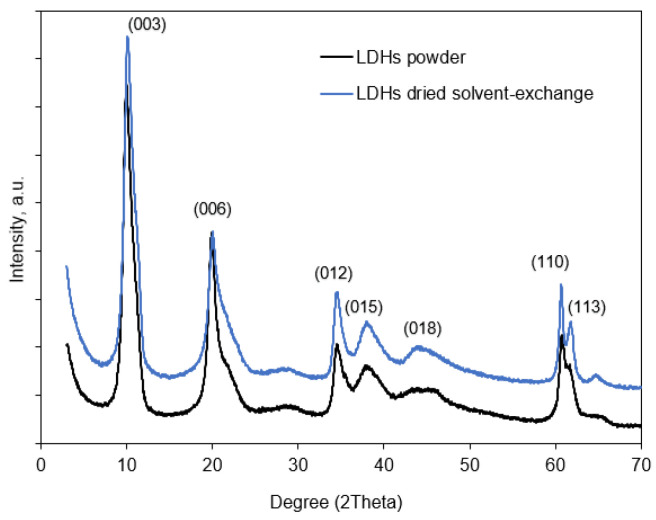
XRD patterns for LDH powder and dried solvent-exchange LDHs.

Figure 5 shows the XRD patterns of dried xylene-treated LDH particles and LDH powder. The resulting diffractograms exhibit characteristic reflections typical of LDH structures. The most intense peak observed at a diffraction angle of approximately 10° corresponds to the (003) reflection (Miller indices). This reflection is a direct consequence of the periodic stacking of the brucite-like metal hydroxide sheets along the c-axis. In this study, the basal spacing was determined to be 8.72 Å (*d*_003_ = c/3). By subtracking the typical thickness of a Mg-based hydroxide layer (4.77 Å), the interlayer gallery height (or interlayer) distance was calculated to be 3.95 [39]. Additionally, the higher-order reflection at 20° is assigned to the (006) plane, further confirming the well-layered architecture of the materials, while the peak around 61° corresponds to the (110) plane, reflecting the in-plane ordering within the hydroxide sheets.

### 3.2. FTIR Spectra Analysis of Epoxy/LDH Nanocomposites

**Figure 6:**
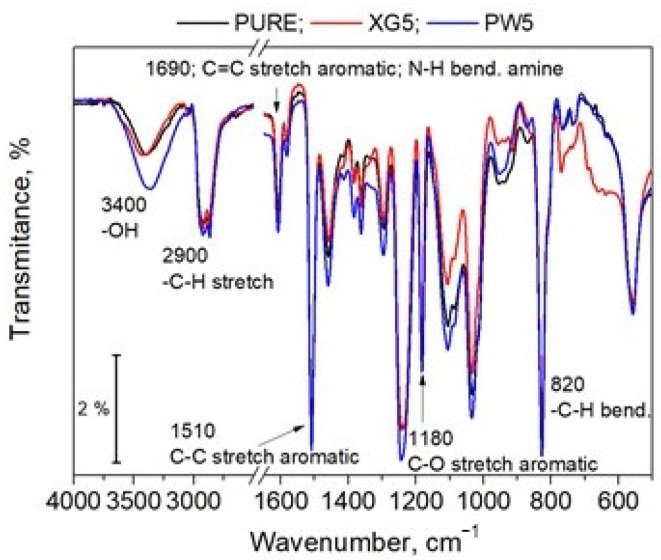
FTIR spectra of pure epoxy and epoxy/LDH nanocomposites with XG and PW particles at 5 wt.%.

Figure 6 shows the FTIR spectra of pure epoxy and epoxy/LDH nanocomposites with 5 wt.% of XG or PW. The key focus was to examine the retention of hydroxyl groups and the preservation of the epoxy structure. The FTIR spectra of the filled nanocomposites closely resemble those of the pure epoxy matrix.

The major difference was detected in the -OH stretching region (stretching vibration detectable at 3300–3600 cm^−1^), where notable differences were observed between the nanocomposites with PW5 and XG5 particles. The PW5 nanocomposite showed increased -OH absorption intensities. It is assumed that the penetration and thus the information depth of the IR radiation did not change with the addition of LDH particles during FTIR measurements. Therefore, the increase in -OH intensity can be attributed to the signal of the LDH hydroxide-base structure and water physisorbed/intercalated between the layers.

In contrast, the spectrum of XG5 nanocomposites showed the same intensity of the -OH group as pure epoxy. This is a result of the repeated replacement of water with organic solvents (ethanol, acetone, and finally xylene), which caused the amount of water present in LDHs to gradually decrease. The residual organic solvents in XG led to changes in the FTIR baseline. Despite these changes in the -OH region between pure epoxy and conventionally filled systems, the LDH-related absorptions are largely masked by the dominant epoxy matrix. Due to strong spectral overlap, it is not possible to analyse even the fundamental vibrations of NO_3_^−^ from the LDH structure (1380 cm^−1^) or Al(Mg)-O fundamental vibrations (600–700 cm^−1^) [40] as the filler content was below 5 wt.%.

It should be noted that no distinct absorption bands were observed in the FTIR spectra that could be directly attributed to Disperbyk 180 or residual xylene in the cured epoxy nanocomposites. The cured epoxy network, formed from bisphenol A epoxy resin and isophorone diamine hardener, exhibits complex and overlapping absorption bands associated with C–O, C–N, O–H, N–H. Consequently, the characteristic bands of low-concentration Disperbyk 180 cannot be reliably distinguished from those of the epoxy matrix. It is also likely that any residual xylene that may remain in the XG5 samples is very low, and its C–H vibrations overlap with those of the cured epoxy network.

### 3.3. Mechanical Properties of Epoxy/LDH Nanocomposite

**Figure 7:**
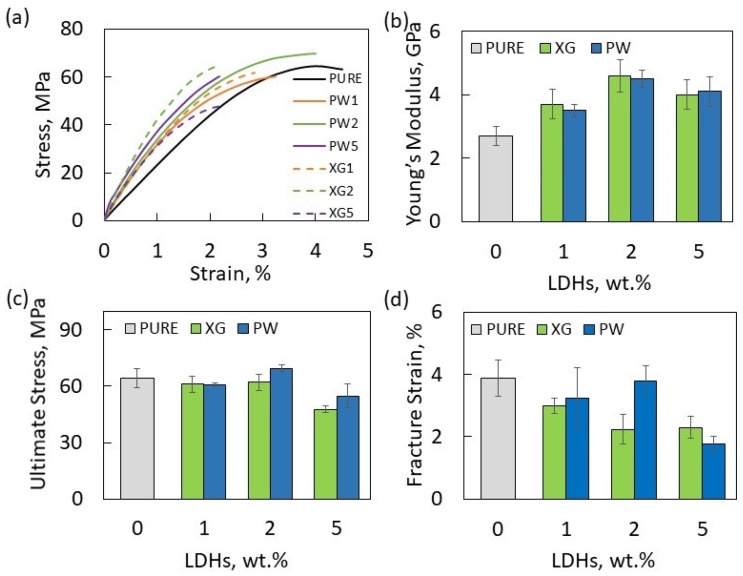
Comparison of tensile properties of epoxy/LDH nanocomposites prepared using solvent-exchange LDHs and dried LDH powder with dispersant: typical tensile curves (**a**); Young’s modulus (**b**); ultimate stress (**c**); fracture strain (**d**).

Figure 7 presents the tensile test results, illustrating the typical tensile curves, dependence of Young’s modulus, ultimate stress, and fracture strain on LDH loading and the method of nanocomposite preparation. The incorporation of LDHs consistently increased Young’s modulus across all examined LDH contents (Figure 7b). With the addition of 1 wt.% LDHs to the epoxy using either method, the modulus increased by approximately 1.3 times, and with 2 wt.% LDHs, it increased by as much as 1.7 times. However, at 5 wt.% LDHs, the modulus no longer increased and even a slight decrease was observed, although it remained 1.5 times higher than that of pure epoxy.

The addition of LDHs had minimal effect on ultimate strength (Figure 7c). When 1 and 2 wt.% LDHs were added, the strength of the nanocomposites produced by both methods remained nearly equivalent to that of pure epoxy, with variations falling within the confidence intervals, except for the PW2 group, where a slight (9%) increase in strength was observed. However, at 5 wt.% LDH loading, the strength decreased noticeably, by 25% in the case of XG samples and 15% for PW. The incorporation of LDHs reduced fracture strain in all cases investigated (Figure 7d). While no clear trend emerged, the fracture strain decreased on average by half when 5 wt.% LDHs was added, regardless of the preparation method.

Furthermore, the results of the mechanical tests did not reveal any plasticising effect that could be attributed to residual xylene. On the contrary, the increased Young’s modulus and decreased fracture strain (Figure 7) indicate that the reinforcing effect of LDH remained dominant, making the material stiffer and less deformable.

To explain the mechanical performance, SEM and EDS characterisation of the epoxy/LDH nanocomposite was employed, which is presented in the following section.

**Figure 8:**
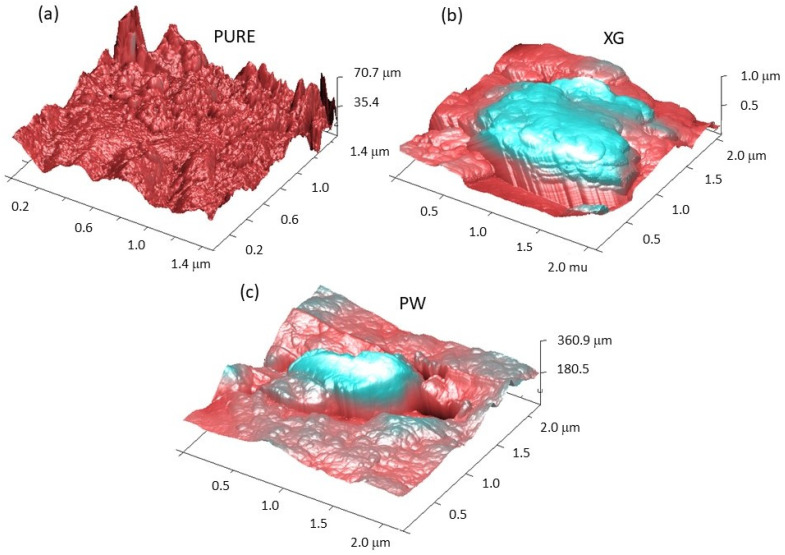
The 3D AFM maps of the topography and map of the mechanical property (slope of the force–distance spectroscopy) of cryogenic cuts of nanocomposites PURE (**a**), XG2 (**b**), and PW2 (**c**) for LDH lamellae oriented parallelly to the surface. The topography is shown by height in the 3D model, and the mechanical stiffness value is represented by the intensity of the blue to red colour of each point.

**Figure 9:**
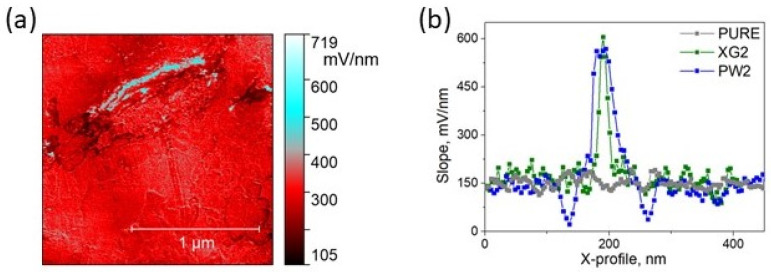
AFM map of the mechanical property (slope of the force–distance spectroscopy) detected on the cryo-cut of the PW sample (**a**) and mechanical property (i.e., slope of the force–distance spectroscopy) profiles for PURE, XG2, and PW2 samples (**b**) for LDH lamellae oriented perpendicular to the surface.

Direct contact measurement of the mechanical properties in the matrix–filler interphase of epoxy/LDH nanocomposites was performed via AFM based on nanomechanical mapping. The samples were prepared using cryo-cutting (cutting temperature −90 °C) to reveal the individual lamellae within the material. Due to the random orientation of the lamellae inside the composite, their behaviour was compared depending on orientation: (i) for lamellae oriented parallel to the cut surface (Figure 8) and (ii) for lamellae perpendicular to the cut surface (Figure 9) in nanocomposite samples with 2 wt.% of LDHs. For comparison, the surface of the epoxy without LDH lamellae is also shown, which exhibits a homogeneous surface with uniform mechanical stiffness [41,42,43,44] as a sum of noise, the influence of topography after cutting, and other effects (Figure 8a).

The nanocomposites with XG2 and PW2 show lamellae with lateral dimensions exceeding 1 µm, protruding above the cut surface. In the XG2 sample, the LDH lamellae appeared topographically elevated and mechanically stiffer (blue/white) compared to the less stiff epoxy matrix (red colour). However, in the case of PW lamellae, their surroundings exhibit significant local decrease in stiffness (and also adhesion) and a local decrease in topography around/on the edges of the lamellae (dark colour in Figure 8c).

In the case of lamellae oriented perpendicular to the cryo-cut (ii), the lamellae appear as mechanically stiffer regions (white–blue colour represents a higher slope in the force-spectroscopy mode of the AFM [43]) with a 1D structure (white line in Figure 9a). Different lamella thicknesses can be detected in the cuts (profiles of mechanical behaviour in Figure 9b), where the XG2 sample shows an increase in stiffness with a half-width of <18 nm, and the surroundings of the lamellae exhibit minor stiffness fluctuations at the noise level in the pure epoxy sample. In contrast, the LDH lamella in the PW2 sample has a significantly wider cross-section (half-width up to 35 nm), and additionally, the interfacial region between epoxy and the lamella shows a local decrease in stiffness (Figure 8c), indicating reduced interfacial adhesion.

This is in good agreement with the results of FTIR analysis, which demonstrated a reduction in the content of -OH groups in XG5 due to gradual substitution with organic solvents well compatible with epoxies. Conversely, the PW2 sample shows a similar lateral size but higher lamella thickness compared to LDHs in the XG2 sample. In addition, the presence of a larger amount of -OH groups reduces compatibility with epoxy, subsequently causing lower macroscopic Young’s modulus (see Figure 7b for samples with 2% LDHs) and reduced nanocomposite compatibility (see Figure 8).

### 3.4. Fractographic Analysis of Epoxy/LDH Nanocomposites

**Figure 10:**
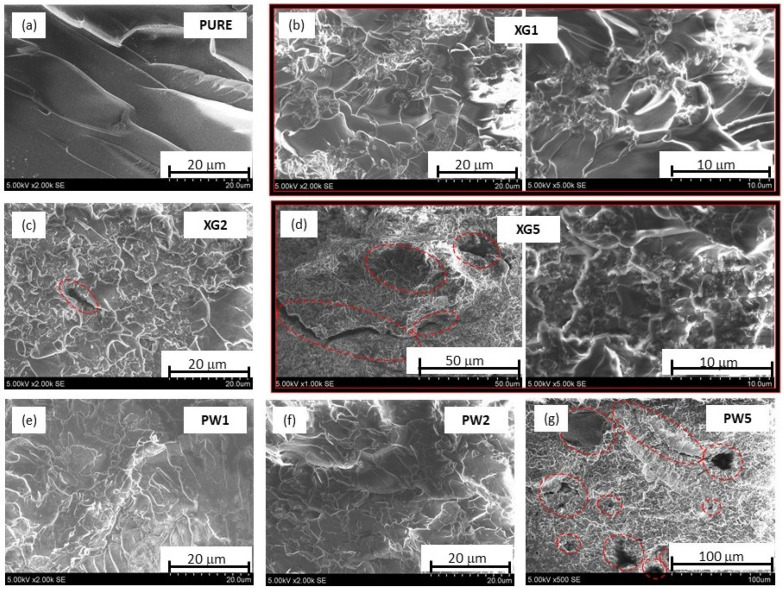
SEM of fracture surfaces of tensile samples: pure epoxy (**a**); epoxy/LDH nanocomposite—solvent-exchange method for 1 (**b**), 2 (**c**), and 5 (**d**) wt.% LDHs; epoxy/LDH nanocomposite—drying method for 1 (**e**), 2 (**f**), and 5 (**g**) wt.% LDHs.

The SEM images revealed clear differences between the fracture surfaces of pure epoxy and epoxy filled with LDH nanoparticles (Figure 10). In pure epoxy (Figure 10a), the fracture surface displayed sparsely distributed, uniformly oriented crack lines, indicative of a smooth and homogeneous failure pattern, typical of brittle fracture. In contrast, the epoxy/LDH nanocomposites exhibited more irregular, multidirectional crack lines concentrated around the nanoparticle regions (Figure 10b–g). This suggests that the LDH nanoparticles disrupted the fracture path, causing crack deflection and more complex failure mechanisms due to stress concentrations around the nanofillers.

For both XG and PW samples, the cracks in epoxy/LDH nanocomposites developed in a similar manner. At 1 wt.% LDH loading (Figure 10b,e), the density of crack lines increased compared to pure epoxy, although not excessively. At 5000× magnification (Figure 10b), it was evident that the LDH nanoparticles concentrated these cracks. As LDH loading increased, the crack line density increased as well. At 5 wt.% LDH, areas with indentations, protrusions, or grooves were identified, likely indicating the presence of defects. The SEM images of 1 and 2 wt.% LDH loading showed relatively good homogeneity and defect-free surfaces, which explains the mechanical performance observed in Figure 7.

The further examination of the SEM fracture surfaces in Figure 7c suggests that the observed depressions are primarily associated with the XG phase rather than general processing defects. The formation of these depressions can be attributed mainly to the pull-out of XG5 aggregates during tensile deformation. At 5 wt.% loading, XG aggregates are formed. Under tensile loading, partial debonding and subsequent pull-out of XG aggregates may occur, leaving void-like features on the fracture surface.

In addition, PW retained small amounts of adsorbed, or interlayer water, as was mentioned previously in the FTIR analysis. During curing, these residual species could contribute to the formation of micro-voids (Figure 10g). Therefore, the increased cavity formation observed at higher PW loading is likely related to a residual volatile species.

**Figure 11:**
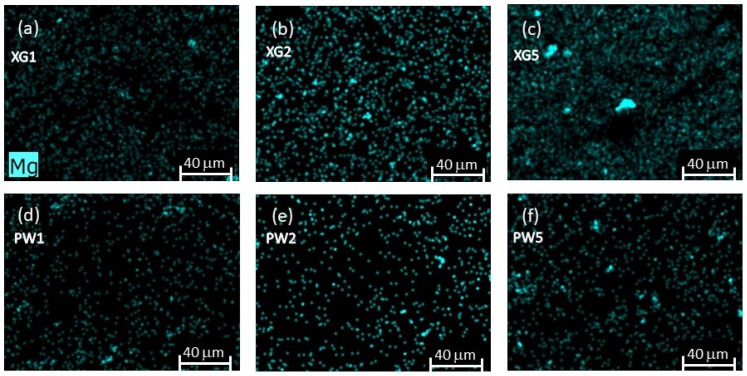
Magnesium (Mg) maps of SEM/EDS analysis of fracture surfaces of tensile samples: epoxy/LDH nanocomposite—solvent-exchange method for 1 (**a**), 2 (**b**), and 5 (**c**) wt.% LDH loading; epoxy/LDH nanocomposite—drying method for 1 (**d**), 2 (**e**), and 5 (**f**) wt.% LDH loading.

To further understand these phenomena, EDS analysis was conducted on the same samples which were examined by SEM to map the chemical elements on the fracture surface. Elemental maps of magnesium (Mg) for all six sample groups were plotted to determine if LDH particles were homogeneously distributed through the epoxy matrix (Figure 11). It can be seen that, unlike at 1 and 2 wt.% loading, larger and more numerous particle aggregates formed at 5 wt.% LDHs, to the same extent in both XG and PW samples. These aggregates acted possibly as defects, creating regions where stress was unevenly distributed, reducing the nanocomposite’s ability to resist deformation. This led to a decrease in both Young’s modulus and tensile strength.

## 4. Discussion

The FTIR analysis reveals that the spectra of pure epoxy dominate the profiles of all filled systems due to the intense absorption bands of the epoxy matrix. These bands overlap and mask many LDH-specific features. However, subtle differences in absorption intensities between XG5 and PW5 samples indicate that the preparation method of LDH influences the -OH content, most likely due to replacement of water with organic solvent molecules in XG5, and ultimately, the performance of the nanocomposites.

Therefore, the comparison presented in this work should be interpreted as a comparison between two practically relevant LDH processing routes: solvent-exchanged LDH slurry and conventionally dried LDH powder processed with a wetting additive. The possible influence of the wetting additive and the different masterbatch component on curing kinetics cannot be fully separated in the present experimental design. However, the FTIR spectra did not indicate major changes in the epoxy network structure, while the observed mechanical and AFM nanomechanical differences were mainly associated with LDH dispersion, residual hydroxyl/water content, and interfacial behaviour.

The significant reduction in hydroxyl groups (-OH) and water content in xylene-treated LDHs facilitated better interfacial interactions with the epoxy matrix. The AFM nanomechanical mapping shows that the interfacial properties of the epoxy/LDH nanocomposites with XG were similar to those of the epoxy matrix. On the other hand, nanocomposites with hydrophilic PW particles with higher water content form regions with low Young’s modulus at the epoxy/LDH interphase. Therefore, surface modification of the LDHs during solvent-exchange treatment leads to stronger interactions between XG and epoxy. Lower water content in XG contributed to good dispersion, defect-free surfaces, and improved mechanical (Young’s modulus) properties, particularly at low (1 and 2 wt.%) LDH loadings. This finding aligns with the SEM and SEM/EDS Mg maps, where the epoxy/XG nanocomposites contained fewer particle aggregates and showed a homogeneous distribution. The addition of LDH nanoparticles likely reinforced the matrix by restricting polymer chain mobility, increasing stiffness and resistance to deformation. Consequently, the Young’s modulus of epoxy/LDH nanocomposites with low particle loading (1 and 2 wt.%) increased.

XRD results confirm that both xylene gel-like solution and powder form LDHs retained their characteristic layered structure. Intense (003) and (006) diffraction peaks at ~10° and ~20° indicate a well-preserved periodic layer arrangement, while the (110) reflection at ~61° confirms the ordered structure of the hydroxide layers. Such a well-organised layered structure acts as a stiffening filler.

The tensile strength of epoxy/LDH nanocomposites with 1 and 2 wt.% particle loading remains unchanged compared with pure epoxy due to the influence of different competing factors. At low LDH loadings, the primary influence on tensile strength is determined by the presence of rigid particles in the matrix, rather than by differences in the interfacial region. The rigid particles restricted the mobility of the polymer chains. On the one hand, LDH nanoparticles reinforced the matrix and promoted crack deflection, delaying failure onset. The observed multidirectional crack lines by SEM suggest improved stress distribution, which could maintain or slightly enhance tensile strength. On the other hand, stress concentrations around the nanoparticles could initiate microcracks, potentially reducing tensile strength compared to pure epoxy. The irregular crack patterns indicate that cracks formed around the nanoparticles, reducing the material’s ability to withstand higher loads before failure. Thus, the overall impact on tensile strength depended on the balance between reinforcement and premature crack initiation. These considerations explain why, at 1 and 2 wt.% LDH loading, the Young’s modulus increased while the ultimate stress remained nearly unchanged.

At 5 wt.% of LDHs, particle agglomeration and the formation of defects in the epoxy matrix were the governing factor. Despite better interfacial interaction in XG, a high LDH concentration still reduced strength and fracture strain.

A preprint version of this manuscript has previously been made available on SSRN [45].

## 5. Conclusions

In this work, a solvent-exchange method was proposed for the preparation of epoxy/Mg-Al/NO_3_ LDH nanocomposites, and their mechanical properties were compared with those of nanocomposites produced via a conventional method based on drying of LDH slurry.

AFM images acquired in topography mode showed that the aspect ratio of the solvent-exchange LDH particles exceeded 1000. XRD analysis indicated similar structural features for LDHs processed by the two above-described methods. The FTIR measurements performed on nanocomposites showed results consistent with decrease in water content of solvent-exchanged LDHs, which may be beneficial for improving compatibility with hydrophobic polymer systems.

Macromechanical characterisation demonstrated that epoxy/LDH nanocomposites produced by both methods exhibited similar mechanical behaviour. Young’s modulus increased significantly at lower LDH loadings (1–2 wt.%) with an increase of approximately 30–70%, indicating enhanced stiffness. The ultimate tensile strength remained largely comparable to that of pure epoxy, while fracture strain decreased across all LDH loadings, indicating increased brittleness with LDH incorporation. At higher LDH contents, a reduction in tensile strength of approximately 15–26% was observed, attributed to particle aggregation, as evidenced by SEM/EDS.

AFM nanomechanical mapping also showed that, due to higher levels of water content on the LDH particle surfaces, nanocomposites incorporating LDH powder formed a wide, low-stiffness interfacial region between the epoxy matrix and LDHs. In contrast, nanocomposites prepared using solvent-exchanged LDH particles via the proposed method exhibited interfacial characteristics more closely resembling those of the epoxy matrix itself.

This study showed that epoxy/LDH nanocomposites prepared using the solvent-exchange method have macromechanical properties comparable to those of composites produced by conventional method, while offering improved micromechanical properties due to a reduction in water content.

The improved interfacial characteristics achieved by the solvent-exchange route may be advantageous for structural and multifunctional composite applications. In addition, the environmentally benign nature of LDHs and their potential flame-retardant and corrosion-protective functions make this approach promising for the development of more sustainable composite systems. Further work should focus on optimisation of processing conditions and evaluation of additional functional properties.

## Figures and Tables

**Table 1:** Nanocomposite sample coding.

Group	PURE	XG	PW
Material description	Pure epoxy	LDH/xylene dispersion gel	LDH powder + wetting additive
Preparation route	NA	I	II
LDH content, wt.%	0	1, 2, 5	1, 2, 5
Sample codes	PURE	XG1, XG2, XG5	PW1, PW2, PW5

## Data Availability

Data are contained within the article. Further inquiries can be directed to the corresponding author.
